# From Restriction to Intuition: Evaluating Intuitive Eating in a Sample of the General Population

**DOI:** 10.3390/nu16081240

**Published:** 2024-04-21

**Authors:** Lorenzo Lucherini Angeletti, Maria Chiara Spinelli, Emanuele Cassioli, Eleonora Rossi, Giovanni Castellini, Giulietta Brogioni, Valdo Ricca, Francesco Rotella

**Affiliations:** 1 Psychiatry Unit, Department of Health Sciences, University of Florence, Largo Brambilla, 3, 50134 Florence, Italy; lorenzo.lucherinibargelliniangeletti@unifi.it (L.L.A.); maria.spinelli@stud.unifi.it (M.C.S.); emanuele.cassioli@unifi.it (E.C.); giovanni.castellini@unifi.it (G.C.); valdo.ricca@unifi.it (V.R.); 2 The Royal’s Institute of Mental Health Research, University of Ottawa, Ottawa, ON K1Z 7K4, Canada; 3 Psychiatry Unit, AOU Careggi Hospital, Largo Brambilla, 3, 50134 Florence, Italy; giulietta.brogioni@unifi.it

**Keywords:** dieting, intuitive eating, eating psychopathology, weight-loss diet, quality of life

## Abstract

Intuitive eating (IE) is a non-dieting approach that promotes listening to internal cues of hunger and satiety, rather than adhering to external dietary restrictions aimed at weight loss. However, the role of IE in dieting behaviors related to weight-loss approaches is still unclear. To address this issue, the aim of this study was to compare IE levels between dieting and non-dieting individuals, exploring the relationship between IE and dieting-related psychological and physical factors. A sample of 2059 females was recruited via social media and self-reported questionnaires were administered to measure IE, eating psychopathology, self-efficacy, and quality of life. Individuals with a history of dieting exhibited lower IE levels, a higher BMI, and a greater eating psychopathology, as well as a reduced self-efficacy and quality of life, compared to non-dieters. IE showed a protective effect against dieting behaviors, with higher IE levels being associated with a lower likelihood of dieting. Additionally, higher BMI and eating psychopathology were predictors of dieting. Promoting IE could represent a relevant clinical target strategy to address disordered eating and enhance overall well-being, underscoring the need for interventions that foster a healthier relationship with food and bodily internal sensations.

## 1. Introduction

Dieting, defined as the deliberate and consistent restriction in calorie consumption aimed at losing or maintaining weight, has garnered significant attention in both public health and psychological research [1,2,3,4]. Successful weight-loss dieting often leads to persistent hunger, particularly when weight loss falls below an individual’s physiological set-point [5]. Long periods of restriction represent an important risk factor for the development of maladaptive eating behaviors [6,7,8] and can lead to both undesirable physical and mental outcomes [9]. Typical-weight individuals who engage in dieting would do so to counteract a strong tendency towards weight gain and disordered eating behaviors, suggesting dieting as a useful indicator of a predisposition to eating psychopathology and a higher body mass index (BMI) in the long term [10].

It has been argued that dieting often involves relying more on cognitive control over eating rather than on physical hunger signals; this reliance makes dieters prone to lose control over their eating, in situations where cognitive control is weakened, such as during times of emotional distress [5,11]. Among the cognitive components pertaining to control over eating, several studies have focused on the role of self-efficacy in dieting. In accordance, it has been found to be related to, and predict changes in, weight and weight-related behaviors, albeit with mixed results across studies [12,13,14,15,16,17,18]. Overall, persistent restrictions and their associated cognitive and emotional dynamics may lead to weight gain (i.e., an increase in BMI) and may heighten the likelihood of developing disordered eating and, ultimately, an eating disorder, with a potential negative impact on the individual’s quality of life [11]. 

To address this issue, some authors have theorized intuitive eating (IE), a non-dieting approach that encourages the recognition and reliance on the body’s internal hunger and satiety signals, as opposed to restrictions or dieting practices [19]. This approach discourages a focus on weight control, as well as judgments towards food and body shape, laying its foundations on an adaptive form of eating behavior, which integrates interoceptive, emotional, and cognitive elements [19,20,21]. IE can be assessed with an instrument (the Intuitive Eating Scale-2/IES-2) that evaluates different aspects of this eating behavior, such as how individuals (i) allow themselves to eat without restrictions, (ii) distinguish between eating for physical and emotional reasons, (iii) rely on internal signals of hunger and satiety, and (iv) choose food according to their bodily needs [22].

Building on the existing literature, recent meta-analyses and systematic reviews provided a nuanced picture of the efficacy of weight-loss diets compared to IE approaches. It has been suggested that, while weight-loss diets can result in short-term weight reduction, they are often unsustainable and may lead to adverse psychological outcomes, including disordered eating patterns [7]. In contrast, interventions focused on IE highlighted an association with improved psychological well-being and a decrease in restrictive eating behaviors [23,24]. Moreover, this approach has also been shown to display a more adaptive eating style, inducing potential long-term benefits for psychological health [25]. Similarly, some evidence has underscored the fact that IE practices correlate with better body image and higher self-esteem, suggesting a protective effect against the development of eating psychopathology [26,27,28,29]. 

Considering one’s internal cues of hunger and satiety is, thus, a critical element in achieving better outcomes, even in weight-loss diets. Evaluating IE within weight-loss programs may not only foster a healthier relationship with food, but also serve as a preventative measure against the acquisition of disordered eating behaviors. In this regard, the evaluation of IE among individuals within the general population, who have either engaged in weight-loss diets or who have not, may represent an important clinical border. Particularly, the potential of IE to predict whether an individual will engage in a weight-loss diet or not, could have significant implications for public health, leading to more focused (psycho)educational programs in subjects with lower levels of IE. 

In this regard, the primary aim of the present study was to compare IE levels between ‘dieters’ (i.e., those who follow or have followed a weight-loss diet in their lifetime) and ‘non-dieters’ (i.e., those who have never followed a weight-loss diet), in a sample collected from the general Italian population. The secondary aims were as follows:

1. To make comparisons between ‘dieters’ and ‘non-dieters’ for the above-mentioned clinical variables that may be related to dieting—BMI, eating psychopathology, self-efficacy, and quality of life.
2. Investigate possible correlations between IE and the same clinical variables in the two groups (‘dieters’ and ‘non-dieters’).
3. Examine whether IE, BMI, eating psychopathology, self-efficacy, and quality of life can predict engagement in weight-loss dietary approaches.

## 2. Materials and Methods

The sample was recruited via two social networks (Instagram and Facebook) with no filter and/or a priori selection of the subjects enrolled. An online questionnaire was posted in September 2022 on 10 Facebook groups and on Instagram by two micro-influencers’ (i.e., less than 100,000 followers) accounts. Inclusion criteria require participants to (i) approve informed consent and (ii) be at least 18 years of age. Participants who failed to completely fill the questionnaire (more than the 95% of the items) were excluded from the sample. A priori power analysis showed that a sample of at least 450 individuals was needed to detect a correlation as small as ρ = 0.20, for a two-tailed Pearson correlation coefficient with α = 0.05 and β = 0.01.

This study was conducted in accordance with the Declaration of Helsinki and was approved by the Institutional Review Board (or Ethics Committee) of the University of Florence (protocol code no. 0173384, 15 July 2022). To participate in the study, all the participants were required to complete an informed consent form. This document comprehensively informed participants about key aspects of the study. Specifically, data were securely informatically stored in computers used for research purposes at the Psychiatry Unit of AOU-Careggi, with strict measures in place to ensure a limited access and the anonymity of collected data. Furthermore, the informed consent outlined the aims of the research, guaranteeing that participants were fully aware of the study’s objectives before contributing their information.

### 2.1. Measures

Demographic information, encompassing variables, e.g., sex, age, and BMI (kg/m^2^), was gathered. Subsequently, a standardized set of questionnaires was administered to each participant as part of the research protocol. We decided to employ self-reported questionnaires for the study, due to their cost-effectiveness, ability to reach a broad and diverse participant base, the rapidity with which they can collect data, and the standardization they offer, ensuring that all participants receive the same questions presented in the same manner, which can enhance the reliability of the gathered information. The following are the self-reported questionnaires used, in addition to the question regarding diet:

- Dieting history:

A simple and straightforward question was asked about having followed a weight-loss diet as a possible index of drive to weight loss, avoidance of weight gain or dieting behavior (“Have you ever followed a weight-loss diet?”—yes/no).

- Intuitive Eating Scale-2 (IES-2):

The Italian version of the IES-2 was administered [30]. This 23-item questionnaire of intuitive eating is rated on a 5-point Likert scale ranging from 1 (strongly disagree) to 5 (strongly agree). The higher the result of the average answers to the questionnaire, the higher the levels of intuitive eating. In addition to the total score, this scale contains four subscales, as follows: (i) “unconditional permission to eat”, which reflects the willingness to give oneself full permission to eat all foods and to remove any judgments or restrictions around eating; (ii) “eating for physical rather than emotional reasons”, which addresses the ability to differentiate between physical hunger and emotional hunger and to primarily eat in response to genuine physical cues; (iii) “reliance on hunger and satiety cues”, which indicates the degree to which one relies on one’s body signals of hunger and fullness to guide one’s eating behaviors; and (iv) “body-food choice congruence”, which assesses the extent to which one’s food choices align with one’s body needs and signals. The Italian adaptation showed an excellent internal consistency (McDonald’s ω = 0.93). In this study, however, we analyzed the IES-2 Total score within the sample.

- Eating Disorder Examination Questionnaire (EDE-Q):

Eating psychopathology was assessed using the Italian version of the EDE-Q [31]. This questionnaire evaluates the presence of behaviors, attitudes, and feelings related to eating behaviors in the past 28 days. The EDE-Q is made up of 28 items, rated on a 7-point forced choice format (0–6), with higher scores reflecting greater severity or frequency. Among them, six items provide the opportunity to specify how many times specific maladaptive eating behaviors were performed in the previous 28 days. The EDE-Q yields a total score and four subscale scores, as follows: (1) restraint, (2) shape concern, (3) weight concern, and (4) eating concern, showing excellent reliability (Cronbach’s α = 0.97) in the Italian adaptation. In the current study, the focus was on examining the total score of the EDE-Q within the sample.

- General Self-Efficacy Scale (GSE):

The Italian version of the GSE was used to assess general self-efficacy [32]. General self-efficacy is considered as a broad and stable confidence in one’s ability to cope with different demanding situations. The 10 items were rated on a scale ranging from 1 (not true) to 4 (exactly true). High test results indicate high levels of self-efficacy. The Italian translation exhibits a Cronbach’s alpha coefficient for the total scale of 0.87, indicating adequate internal consistency.

- 12-Item Short-Form Health Survey (SF-12):

The health-related quality of life is the perceived quality of an individual’s daily life, that is, an assessment of their well-being or lack thereof. This includes all mental and physical aspects of the individual’s life. This construct was assessed with the Italian version of the SF-12 [33], demonstrating a low internal reliability (McDonald’s α = 0.552). The 12 items were assigned to two scores, covering physical or mental aspects of quality of life, in which higher scores indicate a higher quality of life. However, in the present study, the focus was on examining the total score of the SF-12 within the sample.

### 2.2. Statistical Analysis

Data analysis was conducted using JASP (Version 0.18.3) [34]. The primary analysis involved the correlations occurring between the subjects’ scores obtained on the IES-2, EDE-Q, SF-12, and GSE, through Pearson’s correlation coefficients in the total sample and within ‘diet’ and ‘no diet’ groups. Subsequently, an independent samples t-test was used to evaluate the differences reported in the administered scale scores between the ‘diet’ and ‘no diet’ groups. 

The final analysis involved a logistic regression to examine if age, BMI, IES-2, EDE-Q, SF-12, and GSE were able to predict if an individual was part of the ‘diet’ or the ‘no diet’ groups, with ‘enter’ methodology. The logistic regression model was used to estimate the odds ratios, which indicates the likelihood of engaging in dieting behavior for each unit increase in the independent variables, controlling for the other variables in the model. Prior to the regression analysis, preliminary analyses were conducted to ensure no violation of the assumptions of logistic regression, including linearity in the logit, absence of multicollinearity, and adequate sample size [35]. 

The model fit was evaluated using several indicators, including the Chi-square improvement from the null model, McFadden R^2^, Nagelkerke R^2^, Tjur R^2^, and Cox and Snell R^2^ [35]. The model’s predictive accuracy was assessed through a confusion matrix, detailing sensitivity and specificity rates. 

## 3. Results

### 3.1. Descriptive Analysis of the Total Sample

**Figure 1:**
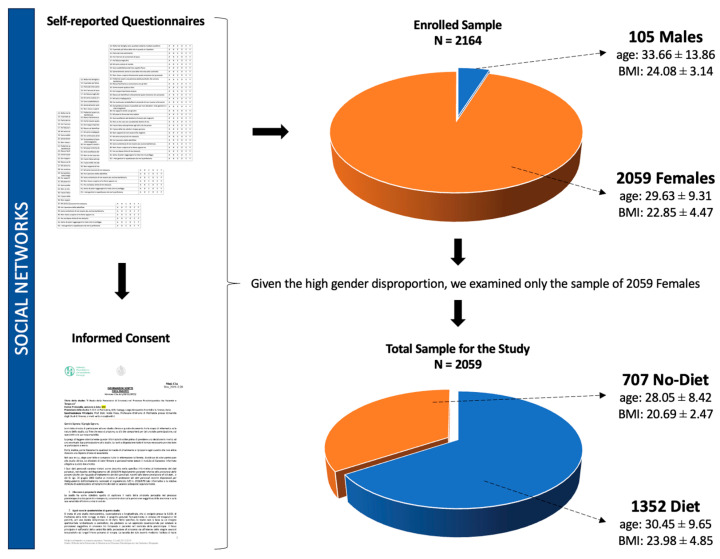
Visual representation of the sample enrollment process and subdivision of the final total sample for the study.

The final sample recruited on social networks consisted of 2164 individuals, divided into 105 males (age: 33.66 ± 13.86; BMI: 24.08 ± 3.14) and 2059 females (age: 29.63 ± 9.31; BMI: 22.85 ± 4.47). Given the high gender disproportion, statistical analyses were performed only on females (Figure 1). Of the 2059 females enrolled, 906 were married or cohabitant (44%), 590 were in a relationship (28.7%), 523 were single (25.4%), 3 were widowers (0.1%), and 37 were “none of the above” (1.8%). Most participants were working full-time (815 women, 39.6%) or were students (785 women, 38.1%); in addition, 195 women were part-time workers (9.5%) and 261 women were unemployed (12.7%). 

### 3.2. Comparisons between “Diet” and “No Diet” Groups

The total sample was divided in two groups, i.e., those (i) who have followed a weight-loss diet in their lifetime (‘diet’, *n* = 1352) and those (ii) who have never followed a weight-loss diet in their lifetime (‘no diet’, *n* = 707) (Figure 1).

Comparisons between the two groups for age, BMI, IES-2, EDE-Q, SF-12, and GSE total scores are reported in Table 1. Subjects included in the ‘diet’ group were older and with a significantly higher BMI. Subjects who had never followed a diet showed higher levels of IE compared to those who had previously followed a diet. Furthermore, the ‘diet’ group showed higher levels of eating psychopathology (EDE-Q) and lower SF-12 and GSE compared to the ‘no diet’ group (Table 1).

### 3.3. Correlation Analyses within “Diet” and “No Diet” Groups

To explore the relationships between IE, BMI, eating psychopathology, self-efficacy, and quality of life within the ‘diet’ and ‘no diet’ groups, simple correlation analyses were performed (Table 2). 

In both groups, the IES-2 displayed a negative correlation with BMI and the EDE-Q. In addition, the IES-2 showed a positive correlation with the SF-12 and GSE only in the ‘diet’ group (Table 2). BMI showed a positive correlation with eating psychopathology in both groups and a negative correlation with the SF-12 only in the ‘diet’ group. Moreover, eating psychopathology showed a negative relationship with the SF-12 in both groups and a negative correlation with the GSE only in the ‘diet’ group. Finally, self-efficacy showed a positive correlation with quality of life in both groups (Table 2).

### 3.4. Logistic Regression Analysis

A logistic regression analysis was performed to examine whether age, BMI, IES-2, EDE-Q, SF-12, and GSE were able to predict a previous dietary attempt in our sample. The analysis revealed significant relationships for all the covariates. Results are reported in Table 3. Higher levels of IE were a protective factor for dieting. In contrast, higher age, BMI, EDE-Q, and SF-12 were significant predictors for dieting. Finally, the GSE failed to predict whether an individual was in the ‘diet’ or ‘no diet’ group (Table 3).

The model demonstrated an overall accuracy of 75.6%. Specifically, the sensitivity of the model, which measures its ability to correctly identify ‘diet’ cases, was 84.0%. This high sensitivity indicates that the model was effective in identifying individuals who were in the ‘diet’ group. On the other hand, the specificity of the model, which assesses its ability to correctly identify ‘no diet’ cases, was 59.6%. This suggests a moderate effectiveness in correctly classifying those not in the ‘diet’ group.

## 4. Discussion

On a sample of 2059 females enrolled via social networks, 1352 had previously followed a weight-loss diet. Our data revealed that these subjects showed a higher age and BMI; a higher level of eating psychopathology; and lower levels of IE, quality of life, and self-efficacy. Furthermore, IE seems to display a protective role for dieting, using a logistic regression approach, correcting for possible confounding factors.

Lower levels of IE may lead individuals to engage in weight-loss diets, as they might rely more on external cues and rules for eating, rather than listening to their own body’s hunger and satiety signals. This hypothesis is confirmed by previous findings, suggesting that decreased IE is associated with a higher susceptibility to dieting and weight control practices, as individuals seek structured dietary guidelines to compensate for their diminished internal regulation [23]. In addition, the fact that ‘dieters’ showed a higher BMI, as well as higher levels of eating psychopathology, underlines the close association between dieting and eating psychopathology in the general population [10]. Individuals with a higher BMI may experience greater body dissatisfaction, leading to an increased motivation to engage in dieting behaviors, to conform to societal standards of beauty [36,37]. Concurrently, elevated levels of eating psychopathology (such as an unhealthy preoccupation with food, body weight, and shape) can exacerbate dieting behaviors, creating a vicious cycle that perpetuates the dieting mentality [38]. With respect to the reported lower levels of self-efficacy and quality of life in the ‘diet’ group, research suggests that individuals on weight-loss diets may experience a lower quality of life and self-efficacy due to the psychological (e.g., potential social isolation) and physical (e.g., restrictions in food choices) distress of dieting [18,39]. Additionally, the constant focus on weight regulation can lead to feelings of failure and frustration when desired outcomes are not met, further impacting their sense of self-efficacy and overall well-being [40].

At a multivariate approach, IE was found to negatively predict dieting (i.e., as IE increased, the likelihood of following a weight-loss diet decreased), correcting for possible confounding factors, such as age, BMI, and eating psychopathology. This supports the hypothesis of a protective role of IE, with respect to the adoption of maladaptive eating behaviors, related to the engagement in diets characterized by external prescriptions and restrictions [24,26,27,41]. Moreover, given the association between IE and interoception (as the perception of internal bodily sensations) [42], this finding resonates with recent research that attributes to reductions in interoception play a role in the etiopathogenesis of eating disorders [43,44,45,46]. 

A higher BMI and higher levels of eating psychopathology also predicted the engagement in a weight-loss diet. This datum is in line with previous findings, highlighting the subsequent risk of disordered eating behaviors and, ultimately, of a full-blown eating disorder [9,10]. Furthermore, age and quality of life were also found to positively predict the likelihood of following a weight-loss diet. In particular, the quality of life outcome should be understood, according to the adjustment, with respect to the other variables, as follows: as quality of life increases (while maintaining the same levels of eating psychopathology, BMI, and IE), the propensity to engage in a diet increases (possibly related to each individual’s personal resources). Lastly, levels of self-efficacy did not predict whether individuals would belong to the ‘diet’ group when considering the effect of other covariates. Nevertheless, this result might rely on the lower scores in self-efficacy observed in ‘dieters’ as compared to ‘non-dieters’. In this regard, it is, thus, conceivable to hypothesize that self-efficacy influences the engagement in a weight-loss diet through IE, potentially explaining the lack of a predictive effect.

The central role of IE in differentiating ‘dieters’ and ‘non-dieters’ holds a strict clinical relevance, considering the many negative physical (e.g., BMI) and psychological (e.g., eating psychopathology) consequences that could derive from repeated dietary attempts [5,11]. The implementation of psychoeducation and interventions that foster a greater connection with one’s body and its feelings of hunger and satiety thus becomes an essential public health issue. In accordance, IE and its aspects have been found to correlate with a higher overall diet quality [47], suggesting that it may be beneficial for promoting healthy eating behaviors, regardless of actual weight-loss. Furthermore, IE has shown positive results in reducing emotional eating, as well as improving and changing eating habits (reducing restrictive behaviors), psychological health, and body satisfaction [29,48]. This may reflect a better relationship of intuitive eaters with their bodies, as well as a greater satisfaction with their weight. Increasing an individual’s body and weight satisfaction has multiple advantages, yet one of the most important would be to make people less likely to engage in dieting behaviors, preventing BMI increases and an individual’s chance of developing disordered eating [8,49,50,51]. 

Within this framework, evaluating IE among those on a weight-loss diet could yield a comprehensive perspective on health that combines psychological well-being with indicators of physical health, such as BMI and body composition. Such an assessment can enhance a clinician’s grasp of a patient’s psychological and physiological condition, providing a more individualized and potentially more effective strategy for addressing eating behaviors and overall health. IE-based psychoeducational programs are essential (even in weight-loss diets), as they aim to improve emotional awareness and reduce instances of emotional eating. By teaching individuals to discern between emotional and physical hunger, these programs can foster healthier eating habits that are aligned with the body’s needs, thereby mitigating the risk of weight cycling and the development of eating disorders often exacerbated by conventional weight-loss diets. Future longitudinal research is required to examine the potential impact of IE interventions (i.e., aimed at fostering an amelioration in the connection with one’s body signals and cues) on enhancing an individual’s body and weight satisfaction, ultimately reducing the likelihood of negative physical (e.g., BMI) and psychological (e.g., eating psychopathology) outcomes that might determine weight-loss dietary attempts.

### Limitations

The present study had some limitations that need to be considered. First, the data relied solely on self-reported questionnaires, which might have been susceptible to social desirability and self-report bias. Second, the cross-sectional nature of the study does not allow causal inferences. Finally, the high gender disproportion of our sample, that led us to perform analyses only on females, does not allow us to consider our data as representative and limits their generalizability.

## 5. Conclusions

The present study evidenced different levels of IE in ‘dieters’ and ‘non-dieters’. Precisely, ‘dieters’ showed lower levels of IE than ‘non-dieters,’ as well as greater complications at the physical (e.g., higher BMI) and psychological (e.g., increased eating psychopathology) levels. Improved connection with one’s body signals and cues (i.e., IE) might, thus, play a protective role in eating psychopathology (also negatively predicting the engagement in weight-loss diets), suggesting that interventions promoting IE may represent an effective prevention/therapeutic option to prevent physical and psychological issues that can burden public health costs.

## Figures and Tables

**Table 1:** Comparison between ‘diet’ and ‘no diet’ groups. IES-2 = Intuitive Eating Scale-2; BMI = body mass index; EDE-Q = Eating Disorder Examination-Questionnaire; SF-12 = Short Form-12; GSE = General Self-Efficacy. * = *p* < 0.05; ** = *p* < 0.001.

	No Diet (*n* = 707)		Diet (*n* = 1352)		*t*	Cohen’s d
	Mean	SD	Mean	SD		
Age (years)	28.05	8.42	30.45	9.65	−5.60 **	−0.26
BMI (kg/m^2^)	20.69	2.47	23.98	4.85	−16.89 **	−0.79
IES-2	3.69	0.61	3.21	0.67	15.79 **	0.73
EDE-Q	0.85	0.89	1.81	1.20	−18.61 **	−0.86
SF-12	33.93	5.79	32.49	6.13	5.14 **	0.24
GSE	2.98	0.49	2.93	0.51	2.00 *	0.10

**Table 2:** Correlation analyses within ‘no diet’ and ‘diet’ groups. IES-2 = Intuitive Eating Scale-2; BMI = body mass index; EDE-Q = Eating Disorder Examination-Questionnaire; SF-12 = Short Form-12; GSE = General Self-Efficacy. * = *p* < 0.05; ** = *p* < 0.001.

**Diet**					
	**1**	**2**	**3**	**4**	**5**
1. IES-2	-				
2. BMI (kg/m^2^)	−0.27 **	-			
3. EDE-Q	−0.65 **	0.23 **	-		
4. SF-12	0.42 **	−0.18 **	−0.52 **	-	
5. GSE	0.33 **	−0.02	−0.30 **	0.46 **	-
**No Diet**					
	**1**	**2**	**3**	**4**	**5**
1. IES-2	-				
2. BMI (kg/m^2^)	−0.26 **	-			
3. EDE-Q	−0.19 **	0.27 *	-		
4. SF-12	0.15	−0.10	−0.51 **	-	
5. GSE	0.08	−0.03	−0.24	0.47 **	-

**Table 3:** Logistic regression analysis of ‘diet’ group. ES-2 = Intuitive Eating Scale-2; BMI = body mass index; EDE-Q = Eating Disorder Examination-Questionnaire; SF-12 = Short Form-12; GSE = General Self-Efficacy.

Predictor	B	SE	OR	Z	Wald	*p*
Intercept	−6.49	0.74	0.01	−8.71	75.94	<0.001
IES-2	−0.25	0.11	0.78	−2.29	5.24	0.022
EDE-Q	0.80	0.08	2.22	10.10	102.06	<0.001
BMI (kg/m^2^)	0.23	0.02	1.26	10.79	116.50	<0.001
SF-12	0.04	0.01	1.04	3.10	9.64	0.002
Age	0.02	0.01	1.02	2.34	5.47	0.019
GSE	0.09	0.12	1.10	0.75	0.57	0.450

## Data Availability

The data presented in this study are available on request from the corresponding author due to privacy and ethical restrictions.
